# Origami Folding by Multifingered Hands with Motion Primitives

**DOI:** 10.34133/2021/9851834

**Published:** 2021-05-30

**Authors:** Akio Namiki, Shuichi Yokosawa

**Affiliations:** Graduate School of Engineering, Chiba University, Chiba, Japan

## Abstract

*Origami*, a traditional Japanese art, is an example of superior handwork produced by human hands. Achieving such extreme dexterity is one of the goals of robotic technology. In the work described in this paper, we developed a new general-purpose robot system with sufficient capabilities for performing *Origami*. We decomposed the complex folding motions into simple primitives and generated the overall motion as a combination of these primitives. Also, to measure the paper deformation in real-time, we built an estimator using a physical simulator and a depth camera. As a result, our experimental system achieved consecutive valley folds and a squash fold.

## 1. Introduction

Manipulation of flexible objects like sheets of paper is a difficult problem for robots. On the other hand, a skilled person can manipulate paper at will, and *Origami*, a traditional Japanese art, is an example of such superior paper manipulation. Achieving such extreme dexterity is one of the goals of robotic technology.

There have been many previous studies on the manipulation of cloth or rope [1, 2]. However, there are not many studies on paper folding. Whereas cloth has no bending elasticity, the paper does, and its properties change as it is folded over and over again. For this reason, origami robots require different manipulation strategies than those used for folding cloth. One of the difficulties in realizing paper folding with robots is that the folding motions are based on multiple fingers' cooperation, and these motions are very complex. Therefore, it is difficult for an operator to teach a robot to generate folding motions and for the robot to realize them. Also, it is necessary to measure the shape and position of the paper in real-time, because the paper changes its shape and position during folding.

Although there have been a number of previous studies on origami folding [3–6], it is challenging to achieve complicated folds. This paper focuses on the system integration of robotic origami using a motion primitive approach. First, we studied the performance required for a folding robot and developed a new general-purpose robot system with sufficient origami capabilities. Next, we decomposed the complex folding motions into individual primitives and generated the overall motion as a combination of these primitives to increase the success rate. To measure the paper deformation in real-time, we built an estimator using a physical simulator and a depth camera. As a result, our system achieved consecutive valley folds and a squash fold.

This paper is an extension of a paper presented at [7], with modifications and new results. This study's originality lies in the development of the first system for achieving high-precision origami and the experimental validation of its performance.

## 2. Related Work

There have been many previous studies on manipulating deformable objects, some of which are reviewed in [1, 2]. On the other hand, there are not many studies on paper folding.

The pioneering work on robotic paper folding is the robot developed by Balkcom and Mason [3, 8]. They developed a robot system specially designed for origami tasks. The robot consisted of an end effector with a large plate and a table with a slot. It made use of this mechanical structure to achieve paper folding. However, its mechanism is so specialized that it is difficult to use it for general origami tasks. Yokokohji's group has developed an origami robot system that consists of four robot arms, and they achieved a folded Tadpole [4]. In their research, the process of the origami task was taught by humans performing origami motions, and complex folding tasks were achieved mainly by focusing on the transition of the human skill to the robot [9, 10]. Christof's group achieved a folding task by using two so-called Shadow Hands [5]. They used a sheet of paper on which many patterns were printed, and the shape of the paper was estimated by recognizing the patterns. Nakashima's group has proposed a motion planner based on a probabilistic model of constraint transitions within human behavior [6].

In the past ten years, our group has developed various types of multifingered hands [11, 12] and achieved various types of tasks, including knotting of a rope [13, 14] and dynamic folding of a sheet of cloth [15]. The essential techniques in this research are extraction of motion primitives from the overall process and the use of visual and force feedback, and we reliably achieved several tasks by using actual robot hands. These techniques have been applied to paper folding, and our purpose is to achieve this task by integrating basic motion primitives [7]. This paper is an extension of our previous work, and by achieving paper folding in an existing system, we verified the validity of the proposed system and the proposed methods via demonstrations.

## 3. Problem Setting

This research is aimed to develop a general-purpose origami robot system that can handle not only simple folds but also intricate folds. There are some problems to overcome due to the difficulty of paper folding.

### 3.1. Complexity of Folding Motions

The robot should automatically generate folding motions for a given diagram of an origami process, as shown in a book. Normally, figures depicting an origami process express the transitions of the state of a sheet of paper. However, the expression is static, and dynamic processes between states are not described. Here, we aim to decompose dynamic processes into basic motion primitives. The motion primitives can be extracted from some typical folding processes, and each primitive should include sensory feedback to deal with unpredicted changes in the sheet of paper. This is discussed in Section 5.

### 3.2. Deformation of Paper

Recognizing the deformation of the sheet of paper is important for achieving paper folding. However, it is difficult to observe the shape precisely because of occlusion by the fingers themselves. We solve this problem by integrating visual feedback of some feature points of the paper and a physical model of the paper. The physical model consists of spring-mass-damper elements in which the dynamic parameters are adjusted to approximate the actual sheet of paper. The physical model deforms according to the visual information and physical simulation, and it is used to estimate the shape of the deformation. This is discussed in Section 4.4.

### 3.3. Range of Working Space of Fingertips

For elaborate origami works, complicated finger configurations are required, and the range of motion of the fingertips should be large. Humans can move their arms and their upper body with the movement of their waist and backbone, which increases the range of motion of the fingers. However, it is not easy to add an upper-body axis, such as the waist, to the robot system. Therefore, we increased the number of degrees of freedom (DOF) of the fingers themselves to 7 to expand the fingers' range of motion. This is discussed in Section 4.1.

## 4. Origami Robot System

**Figure 1:**
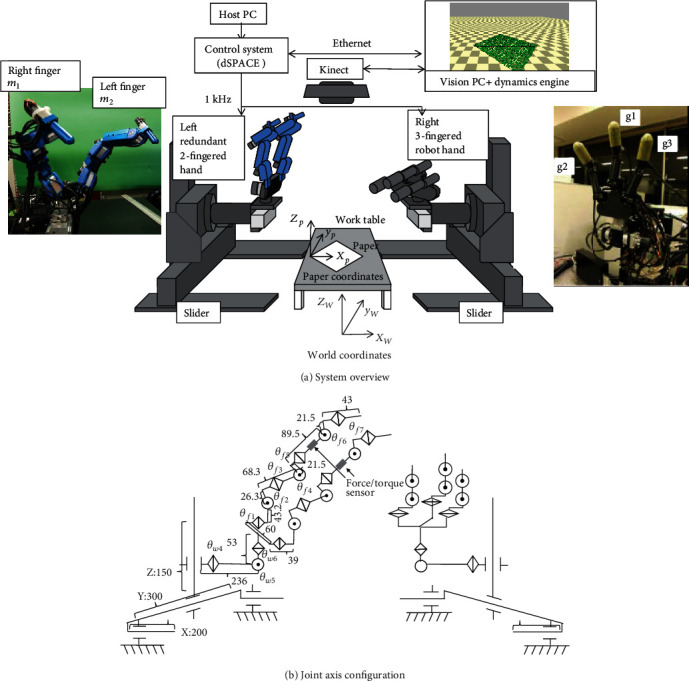
System configuration.

We developed a human-like, dual-armed, multifingered hand system for complex folding motions. The system configuration is shown in Figure 1. Each arm is a 6-DOF manipulator consisting of a linear slider with orthogonal axes and a wrist with three rotational DOF joints. A different type of multifingered hand is attached to the top of each arm.

There is a work table at the center of each arm, and the shape of the origami paper is measured with a depth sensor (Microsoft Kinect) placed above the table. The Vision PC (Core-i7, 3.9 GHz) performs image processing using 2D and depth images obtained from the depth sensor and physical simulation of the paper. The robots are controlled by a real-time controller (DS1006, dSPACE Inc.) with a cycle rate of 1 kHz, and the communication between the PC and the real-time controller is performed asynchronously using UDP protocol.

**Figure 2:**
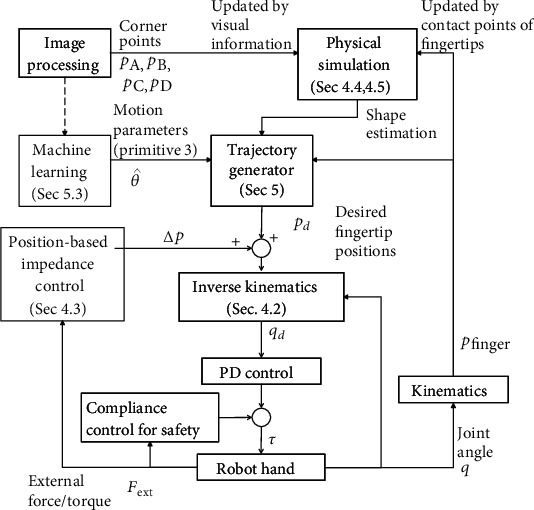
Block diagram of processing.

The process flow of the system is shown in Figure 2. The image processing block sends the 3D position of the corners of the paper to the physical simulator. The physical simulator estimates the shape of the paper in real-time. To update the shape, it uses visual information and the kinematic information of the fingers.

The estimated shape is sent to the trajectory generator, and the desired fingertip positions are calculated. In the turning-up primitive explained in Sec. 5.3, a machine learning method is used to obtain the desired trajectory of the pushing finger. The fingertips' desired trajectories are transformed into those of the joint angles by inverse kinematics, and they are sent to the PD controller. We use position-based impedance control to stabilize the contact force.

### 4.1. Robot Hand and Arm

The system has a robot hand with two redundant fingers at the end of the left arm and a three-fingered robot hand at the end of the right arm. The left two-fingered hand is used for fine manipulation, and the right three-fingered hand is mainly used to hold the paper.

#### 4.1.1. Left Two-Redundant-Fingered Hand

The left hand is a two-fingered robot hand with seven DOFs in each finger. The number of DOFs in each finger is large compared to human fingers or fingers of a typical robot hand, which is useful for realizing a large range of motion. The specifications are shown in Table 1. Harmonic drive actuators (RSF-5A, RSF-3B) are used to prevent backlash, and AU means an actuator unit with RSF-3B. An optical encoder measures each joint angle, and there is a six-axis force/torque sensor between the fifth and sixth axes. Each fingertip has a urethane rubber cap to prevent slipping. Also, a nail tip is added to make a firm crease.

#### 4.1.2. Right Three-Fingered Hand

The right hand is a three-fingered robot hand with four degrees of freedom in each finger. The specifications are shown in Table 2. This hand has the same structure as the high-speed hand proposed in [11], with the addition of a swivel mechanism at the fingertip [12]. Harmonic drive actuators (RSF-5A, RSF-3B) are used to prevent backlash. An optical encoder measures each joint angle, and each joint is equipped with a strain gauge for force measurement. A urethane rubber cap is attached to each fingertip to prevent slipping.

#### 4.1.3. Dual-Arm

The specification is shown in Table 3. Each of the three linear axes is driven by a ball-screw-type linear slider with an HF-KP13B actuator (Mitsubishi Electric). A braking mechanism is provided only on the *z*-axis to hold the axis stationary. Harmonic drive actuators (FHA-14C, FHA-8C, and CSF-8 Harmonic Drive) are used to prevent backlash in the wrist's three axes.

### 4.2. Inverse Kinematics of Left Two-Redundant-Fingered Hand

The two-fingered hand has many degrees of freedom, which complicates the calculation of the inverse kinematics. Here, we use the damped Gauss-Newton method to avoid singular postures [16].

If the joint displacement vector is **q** ∈ **R**^7^, and the error between the desired posture and the current posture is **e**(**q**) = [(**p**_*d*_ − **p**)^⊤^, **α**(**R**_*d*_**R**^⊤^)^⊤^]^⊤^, where **p**_*d*_, **p**(**q**) ∈ **R**^3^ are the desired and current position of the fingertip, **R**_**d**_, **R**(**q**) ∈ *SO*(3) are the desired and current orientation, and **α**(**R**) is the angle-axis vector.

The inverse problem can be solved by minimizing the following cost function:

(1) $$\begin{array}{c} E\left(q\right)=\frac{1}{2}e{\left(q\right)}^{⊤}W_{E}e\left(q\right)⟶\min, \end{array}$$

where **W**_*E*_ = diag{*w*_*E*,*i*_} is positive-definite and diagonal. As a result, the joint angle **q** is obtained by using

(2) $$\begin{array}{c} q_{k+1}=q_{k}+{\left(J_{k}^{⊤}W_{E}J_{k}+W_{N}\right)}^{-1}\left(J_{k}^{⊤}W_{E}e\left(q_{k}\right)\right), \end{array}$$

where *k* is the iteration number, and **J**_*k*_ ∈ **R**^6×7^ is the joint Jacobian. Here, **W**_*N*_ is a damping factor and is defined as $W_{N}=E\left(q_{k}\right)I+{\bar{W}}_{N}$, where ${\bar{W}}_{N}=\mathrm{diag}\left\{w_{N,i}\right\}$ is a small bias to achieve stability around singular postures.

### 4.3. Force Control

For precise manipulation, the force that pushes the paper sheet toward the table should be accurately controlled. In this study, we use a position-based impedance control method [17]. With the vertical direction on the table serving as the *z*-axis, we assume that the target impedance model is written as

(3) $$\begin{array}{c} F_{z}-F_{d}=M_{d}\left(\ddot{z}-{\ddot{z}}_{d}\right)+D_{d}\left(\dot{z}-{\dot{z}}_{d}\right)+K_{d}\left(z-z_{d}\right), \end{array}$$

where *F*_*d*_, ${\ddot{z}}_{d}$, ${\dot{z}}_{d}$, and *z*_*d*_ are the desired values of force, acceleration, velocity, and position, respectively. Here, *M*_*d*_, *D*_*d*_, and *K*_*d*_ are the mass, damping coefficient, and spring constant, respectively.

By discretizing the equation with the sampling time of *T*_*s*_, we obtain the following equation:

(4) $$\begin{array}{c} z_{\left(i\right)}=z_{d\left(i\right)}+\frac{\left(F_{z}-F_{d}\right)+\left(D_{d}/T_{s}\right)\Delta z_{\left(i-1\right)}+\left(M_{d}/T_{s}^{2}\right)\left(2\Delta z_{\left(i-1\right)}-\Delta z_{\left(i-2\right)}\right)}{K_{d}+\left(D_{d}/T_{s}\right)+\left(M_{d}/T_{s}^{2}\right)}, \end{array}$$

where *i* indicates the number of the control step, and ∆*z*_(*i*)_ = *z*_(*i*)_ − *z*_*d*(*i*)_. The value *z*_(*i*)_ is used as the modified desired position on the *z*-axis at step *i*, resulting in the desired impedance property.

### 4.4. Paper Shape Estimation by Physical Simulation

**Figure 3:**
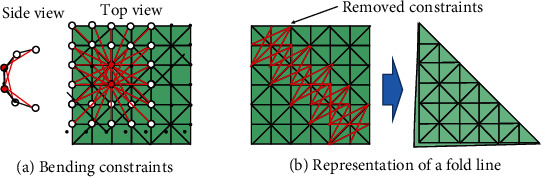
Paper model.

We use a paper model in which many quality points are connected by springs, as shown in Figure 3(a). This is a general model that represents a two-dimensional flexible sheet, like a cloth. However, the paper is characterized by small deformation in the stretching direction and large bending elasticity. Therefore, an additional spring is set up to bridge every one or two quality points, as shown in Figure 3(a). A crease is achieved by removing the spring constant of the link that bridges the quality points on the left and right sides of the corresponding line, as shown in Figure 3(b).

We implemented the simulation by using the soft body function of Bullet Physics [18]. In Bullet Physics, the position-based method (PBD) [19] is used. This is a method of updating each node's position by applying a displacement proportional to the force applied to each node. The accuracy is lower than the force-based method, which uses forward dynamics to update each node's position; however, the computational speed is faster. On the other hand, the deformation's transient response is different from that of the actual paper deformation, and it cannot be used as an estimate of the dynamic response of the actual paper deformation. Therefore, the results of the simulation can only be used in equilibrium.

The larger the number of nodes, the more accurate the estimation becomes, but the computation time becomes enormous. The number of nodes was set to 16 × 16 to achieve real-time processing on the current system. On the other hand, recently improved systems have succeeded in increasing the number of nodes to 64 × 64.

### 4.5. Visual Processing and Updating of Paper Model

We use Kinect 3D vision as an image processing sensor to measure the 3D positions of the four corners of the paper. The four corners of the paper are colored orange, green, yellow, and purple. Each corner's center of gravity is measured in a 2D image from Kinect by performing a color extraction process. The 3D position of each corner is then calculated by mapping the centers of gravity to the depth image obtained from Kinect's depth sensor.

The physical model is updated by using these 3D positions. The corresponding quality point position is set in each corner of the physical model to match the 3D position of the measured corner. The physical simulator is used to find the equilibrium state of the nodes for the given corner positions. The shape of the paper in this equilibrium state is used as the estimated shape of the paper. If the corner is no longer visible, the value measured in the previous step is used.

## 5. Motion Primitives

### 5.1. Extraction of Motion Primitives

#### 5.1.1. Decomposition of Folding Process to Primitives

**Figure 4:**
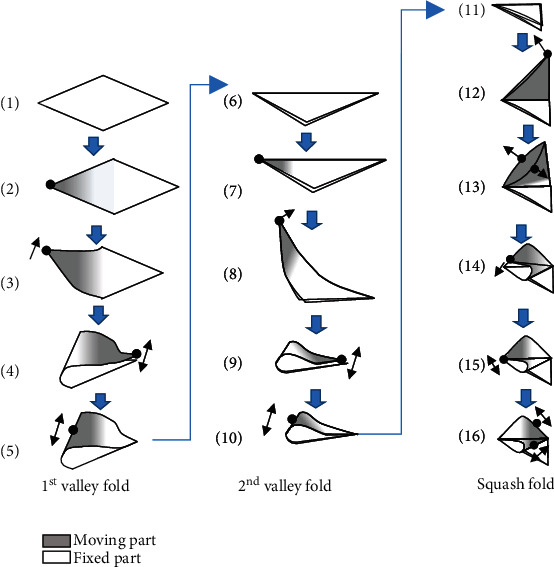
Decomposition of origami folding to primitives.

Based on a sheet of paper's state change, we decompose a folding operation into its basic primitives.  Figure 4 shows the paper state's transition during a folding operation from a first valley fold, through a second valley fold, to a squash fold. In most paper folding processes, only a part of the paper is moved around a corner or an edge. On the other hand, the rest of the paper is fixed in place. The former is called the moving part, and the latter is called the fixed part. For example, (1) shows the paper on the table, and (2) shows that one corner of the paper can be moved.

In some cases, even if the moving part's area remains the same, the way it is moved may change. For example, (3) shows that one of the corners is moved, and then, a turning-up movement is performed. This means that the 3D position and orientation of the corner change. On the other hand, (4) shows an operation to align two corners. Here, the moving part's location is almost the same, but the operation changes to a 2D operation in which the corners are aligned by sliding them on the table.

After (4), the motion is extracted as a primitive when (I) the position of the moving part changes, and (II) the degree of freedom of the moving part changes. Also, (III) when the paper's position or orientation changes, the motion is extracted as a primitive. In these transitions, the method of fixing and moving the paper changes, and the finger placement and movement inevitably change, so it is considered appropriate to extract them as primitives.

#### 5.1.2. Realization of Motion Primitive

**Figure 5:**
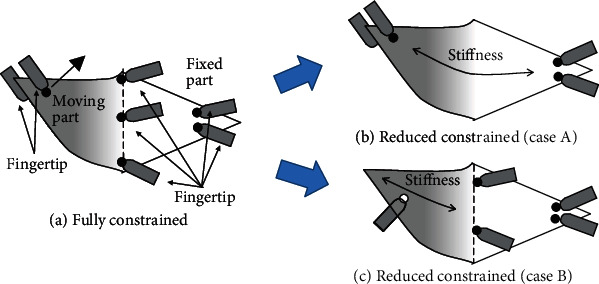
Reduction of constraints.

Next, we consider how to realize the primitives. The moving part must be subjected to sufficient force, and the fixed part must be sufficiently fixed.  Figure 5(a) shows an example of finger placement for the turning-up movement shown in Figure 4(3), assuming that the object is a flexible sheet such as cloth without rigidity. Since the object is flexible, it is necessary to place many constraint points to provide sufficient tension. On the other hand, if the object is paper, we may be able to reduce some of the constraints by using the paper's stiffness and elasticity. In Figure 5(b), the constraint near the crease is removed, and the paper is bent using its bending elasticity. On the other hand, in Figure 5(c), the constraint near the corner is removed, and the paper is bent only by pushing with one finger instead of grasping the corner. In both cases, the same motion can be achieved.

#### 5.1.3. Motion Primitives for Origami Folding

**Figure 6:**
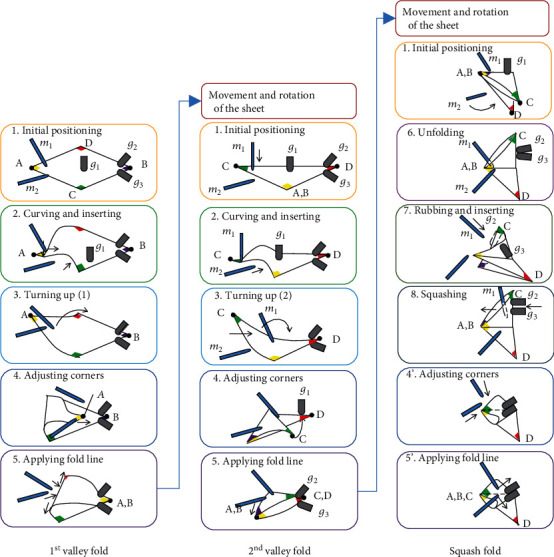
Paper folding sequence and motion primitives.

In a real robot, the number of fingers and the range of movements are limited, so it is necessary to reduce the number of constraints in the kinematics. In this paper, the primitives were decided by trial and error according to the above rule. The extracted motion primitives are shown in Figure 6. The optimization of finger placement can be automated, and this is a future task.

- *Primitive 1: Initial Positioning.* This is a motion primitive that only requires the proper placement of the fingertip positions. It is necessary to estimate the paper state in advance, and the correct initial placement corresponding to the paper state will have a significant influence on the success or failure of the following folding motion. If the initial placement is complex, it is necessary to generate trajectories so that the fingers do not collide with each other
- *Primitive 2: Curving and Inserting*. When a paper sheet is placed on a table, the gap between the table and the sheet is so narrow, making it difficult to insert a finger into the gap. Therefore, it is necessary first to make a gap between the paper and the table. Although various methods to create the gap can be considered, in this study, we adopted a method to create the gap by bending the paper sheet with one hand. By moving the sheet's edges in parallel with the table by a finger movement, the gap is created. The physical simulator is used to estimate the shape of the paper curves. This is explained in Section 5.2.
- *Primitive 3: Turning-Up.* The paper sheet should have sufficient tension during the folding process to prevent wrinkles. Therefore, it is necessary to place a holding finger on the fold line and turn the sheet by gripping the sheet's corner while applying tension to it. However, the number of fingers on the robot hand is limited. Therefore, in the first valley fold, by using the sheet's bending elasticity, the holding finger is not used. On the other hand, the bending elasticity is not uniform in the second valley fold because of the overlap, so the same strategy cannot be used. Therefore, the holding finger (one finger on the left hand) is placed on the fold line, and then, a turning-up movement is performed by pressing the back of the sheet with one fingertip alone. This turning-up motion depends on the contact condition between the fingertip *m*_2_ and the sheet. However, it varies for each trial, and it is not easy to know the condition in advance. To optimize the turning-up motion, we use a machine learning method. This is explained in Section 5.3.
- *Primitive 4: Adjusting Corners.* The turned-up corner is moved by fingertip *m*_2_ to match with the opposite corner. In this primitive, visual feedback by the camera is used
- *Primitive 5: Applying Fold Line.* By using the fingertips *m*_1_ and *m*_2_, a fold line is applied to the paper. The position-based impedance control explained in Sec.  4.3 is used
- *For the squash fold, some different motion primitives are required, as follows.*
- *Primitive 6: Unfolding.* This is the motion of opening the fold line. The two-fingered hand grips the top corner of the sheet and stands the upper part perpendicular to the bottom part
- *Primitive 7: Rubbing and Inserting.* This is the motion of opening the upper part of the sheet to create a gap. First, by rubbing two folded parts of the sheet together with two fingertips of the right 3-fingered hand, a small gap is created. Next, the left robot hand inserts a finger into the gap and expands it. However, the finger's surface is not smooth, and the sheet of paper may get caught on the surface. Therefore, in practice, we attached a stick to the left hand and used it instead of the finger
- *Primitive 8: Squashing.* This is the motion of opening the gap and squashing it. The right hand's two fingertips are placed on either side of the fold line, and the surface is gently squashed
- *Primitive 4': Adjusting Corners.* This primitive is similar to Primitive 4 in the valley folds. The corner is moved by the fingertips to match with the opposite corner. In this primitive, the system uses visual feedback with the camera
- *Primitive 5': Applying Fold Line.* This primitive is almost the same as Primitive 5 in the valley folds. By using the fingertips *m*_1_ and *m*_2_, a fold line is applied to the paper. The position-based impedance control explained above is also used

The system judges the success or failure of a motion by vision, that is, on the basis of each corner's position. If it is difficult to determine the success or failure of an operation using vision, the operation is advanced to a phase where visual recognition is possible, and then, the success or failure is determined.

#### 5.1.4. How to Use Physical Simulator with Motion Primitive

It is difficult to deal with complex shape changes in the current paper model. It is also difficult to simulate large changes in the state of the paper. For example, it is difficult to add crease lines automatically. Furthermore, since the state of the paper is updated using only the corner information, the accuracy of the estimation decreases if the occlusion hides even one corner.

This study used this simulator only when the occlusion was small and sufficient estimation was obtained. It is expected that the success rate of the folding operation will be improved by using the model in all processes, and this is a future issue to be addressed. Specifically, we used this simulator only for (1)–(3) of the first valley fold and (1)–(3) of the second valley fold in Figure 6. In the latter case, we prepared a prefolded model and switched to it. As for the occlusion problem, our recent studies have shown that the estimation accuracy can be improved by measuring the 3D shape of the paper or by adding the edge estimates of the paper [20, 21].

### 5.2. Estimation of Paper Shape in Primitive 2 (Curving and Inserting)

During curving and inserting, it is desired that fingertip *m*_2_ be inserted into the highest point of the generated gap not to be caught on the edge of the sheet. However, it is difficult to accurately detect the shape of the gap by visual sensing because of occlusion by the hands.

**Figure 7:**
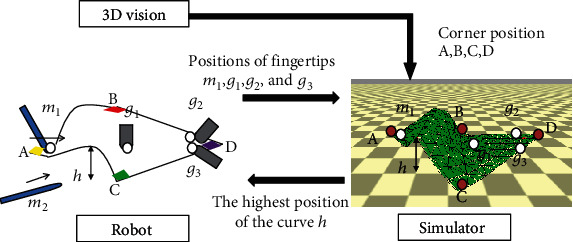
Paper shape estimation in Primitive 2: curving and inserting.

Assuming that there are no slips between the positions of the fingertips and the paper sheet during the curving primitive, the deformation of the paper is estimated based on the physical model, as shown in Figure 7. In addition to the corner information from visual information processing, the positions of the left and right fingertips are given as constraints to improve the accuracy of the paper model's shape estimation. A normal force proportional to the normal distance between the finger and the sheet and a tangential force proportional to the tangential travel distance of the finger from the paper sheet are applied to the nodes around the finger.

The inserting primitive is controlled considering the highest point of the gap simulated in the model.

### 5.3. Motion Correction in Primitive 3 (Turning-Up) of Second Valley Fold

In the second valley fold, the turning-up motion depends on the contact condition between the fingertip and the sheet. One finger is placed on the fold line, and one finger turns up the paper, which may cause slippage between the pushing finger and the paper. Also, because the paper is folded and overlapped, the paper's rigidity is affected, and the direction in which the finger turns up and the direction in which the sheet is folded do not always coincide.

Therefore, we compensate for variations in the turning-up movement by learning the expected value of the relationship between the initial state and the postfolding state. We use least square learning with a *l*2 constraint to compensate for the variations.

#### 5.3.1. Learning of Turning-Up

**Figure 8:**
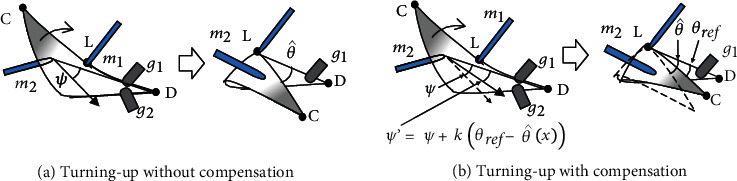
Motion correction in Primitive 3: turning-up.

The relationship between the initial state and the angles of the corners after folding is as shown in Figure 8(a). Let point C be a corner of the paper sheet, point D be the opposite corner, and point L be the fingertip. The input **x** is defined as $x={\left[\overset{\to }{LC},\overset{\to }{LD}\right]}^{T}$, and the output is the angle *θ* between $\overset{\to }{LC}$ and $\overset{\to }{LD}$ after turning-up. The trajectory of the pushing motion of finger *m*_2_ inclined at angle *ψ*.

Assume that the relationship is described as a nonlinear function *f*:

(5) $$\begin{array}{c} \theta =f\left(x,\psi \right)+\varepsilon , \end{array}$$

where *ε* is the Gaussian noise. The finger movement angle *ψ* is assumed to be constant, and the estimate of *θ* from **x** is learned from multiple trials.

We use a Gauss Kernel model for learning, expressed as

(6) $$\begin{array}{c} \hat{\theta }=\overset{N}{\underset{j=1}{\sum }}\alpha_{j}K\left(x,x_{j}\right), \end{array}$$

where **α** = [**α**_1_,⋯,**α**_*N*_]^*⊤*^ is a coefficient parameter vector and *K*(**x**, *c*) is a kernel defined as

(7) $$\begin{array}{c} K\left(x,c\right)=\exp\left(-\frac{{\left\|x-c\right\|}^{2}}{2h^{2}}\right), \end{array}$$

where **c** is the center of the kernel and *h* is its width.

When the inputs for training are {**x**_*i*_}_*i*=1_^*n*^, the squared error *J*_*LS*_(**α**) is defined as

(8) $$\begin{array}{c} J_{LS}=\frac{1}{2}{\left\|\Phi \alpha -\theta \right\|}^{2}, \end{array}$$

where **θ** = [*θ*_1_, ⋯,*θ*_*n*_]^*⊤*^ is the output vector corresponding to the training inputs, and

(9) $$\begin{array}{c} \Phi =\left[\begin{array}{ccc} K\left(x_{1},x_{1}\right) & \cdots & K\left(x_{1},x_{n}\right)\\ \vdots & \ddots & \vdots \\ K\left(x_{n},x_{1}\right) & \cdots & K\left(x_{n},x_{n}\right) \end{array}\right]. \end{array}$$

To prevent overfitting, we use least-square learning with an *l*2 constraint. The estimated parameter $\hat{\alpha }$ is calculated as

(10) $$\begin{array}{c} \hat{\alpha }=\arg\underset{\alpha }{\min}\left[J_{LS}\left(\alpha \right)+\frac{\lambda }{2}{\left\|\alpha \right\|}^{2}\right]={\left(\Phi +\lambda I\right)}^{-1}\theta . \end{array}$$

where *λ* is a parameter that controls the importance of the regularization term. By substituting $\hat{\alpha }$ into Eq. (6), we obtain the estimated angle $\hat{\theta }$.

#### 5.3.2. Motion Correction of Turning-Up

Based on the estimates in the previous section, the finger angle *ψ* is corrected so that *θ* matches the desired value *θ*_ref_, as shown in Figure 8(b). *θ*_ref_ is usually set to 0 to match the two corner points. The difference between *θ*_ref_ and the estimate $\hat{\theta }$ is given by

(11) $$\begin{array}{c} \theta_{\text{ref}}-\hat{\theta }=\theta_{\text{ref}}-f\left(x,\psi \right)≃\theta_{\text{ref}}-f\left(x,\psi^{\prime }\right)-\frac{df}{d\psi }\left(\psi -\psi^{\prime }\right)=\frac{df}{d\psi }\left(\psi -\psi^{\prime }\right), \end{array}$$

where *ψ*′ is the corrected angle of *ψ*. Here, *f*(**x**, *ψ*) is approximated by a Taylor expansion around *ψ*′.

As a result, the inclined angle *ψ* is corrected as

(12) $$\begin{array}{c} \psi^{\prime }=\psi +k\left(\theta_{\text{ref}}-\hat{\theta }\right), \end{array}$$

where *k*^−1^ = *df*/*dψ*. Since it is difficult to estimate the exact value of *k*, we assumed that *k* is a constant value.

## 6. Experiment

### 6.1. Initial Setting

**Figure 9:**
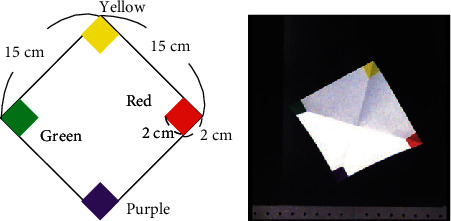
Paper sheet.

We used commercial A4-size sheets of printer paper and cut them into square shapes whose side length was 15 cm, as shown in Figure 9. The four corners of each square sheet were colored green, yellow, purple, and orange, respectively. The shape of the colored part at each corner was a 2 cm square.

### 6.2. First Valley Fold

The first valley fold was verified, and it was successfully achieved in all ten trials. The distance between the two vertices A and B after folding is shown in Table 4. The minimum error was 0.54 mm, and the average error was 2.70 mm, indicating that highly accurate folds were achieved.

**Figure 10:**
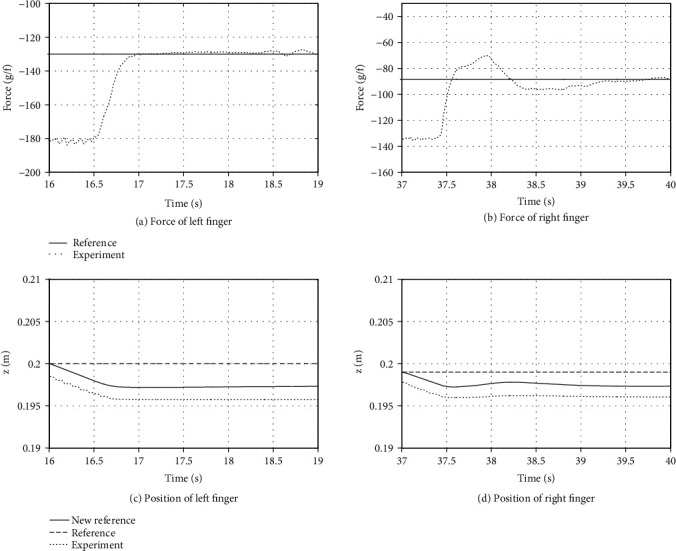
Force and position of fingertips of left 2-fingered hand.

Figures 10(a) and 10(b) show the response of the force control in the *z*-axis direction of each fingertip of the left hand during the deflection motion. The right finger pushed and deflected the paper, and a large force was applied at the beginning of the movement, but it gradually stabilized.

The left finger was used to hold the paper in place, and stable force control was achieved.

The responses of the position control during the deflection motion are shown in Figures 10(c) and 10(d). The measured values followed the target values. Here, the new reference was the new target value generated by the position-based impedance, and the position target value changed by the amount of the pushing force. The gap between the target value and the measured value was caused by insufficient gravity compensation, but it was not a severe problem in this experiment.

**Figure 11:**
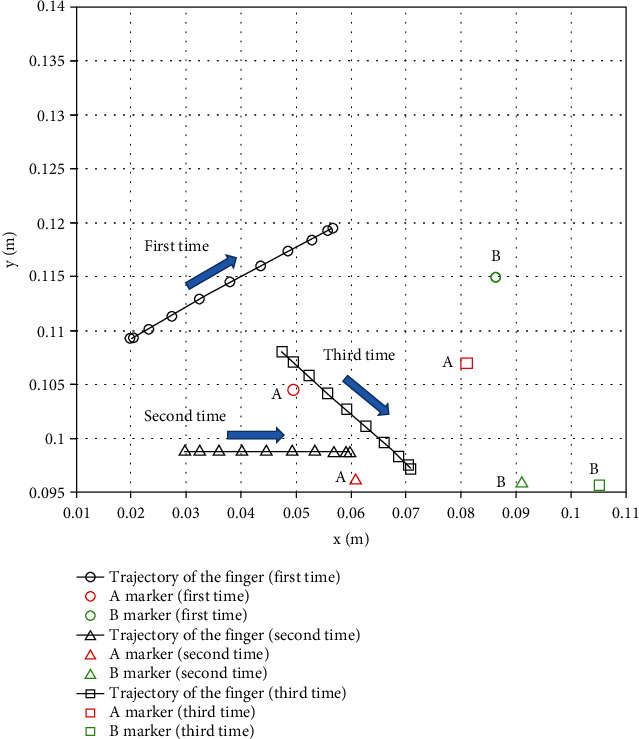
Trajectory in primitive 4: adjusting corners.

The positions of the corner markers and the corresponding finger trajectories are shown in Figure 11 for three of ten motions in Primitive 4 (adjusting corner). It is shown that the trajectory was corrected by visual feedback when the paper's initial position was changed.

#### 6.2.1. Estimation of Paper Shape in Primitive 2 (Curving and Inserting)

**Figure 12:**
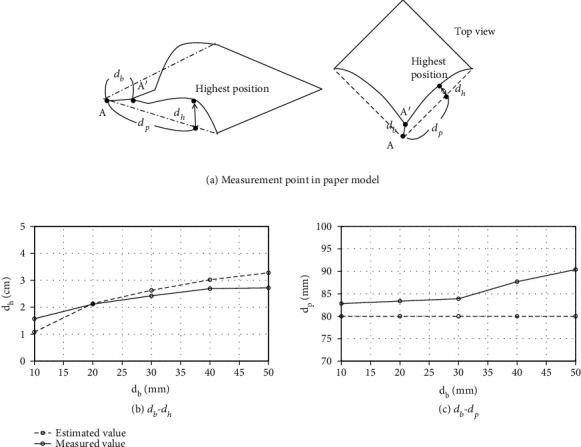
Result of estimation of paper shape.

We examined the difference between the shape of the physical model and the actual paper during Primitive 2 (curving and inserting) in the first valley-fold. The parameter settings are shown in Figure 12(a), where *d*_*b*_ is the distance between the corners, *d*_*h*_ is the height of the highest point of the deflection, and *d*_*p*_ is the distance between the initial corner point and the highest point. We measured *d*_*h*_ and *d*_*p*_ for *d*_*b*_ as 10, 20, 30, 40, and 50 mm in both the actual paper and the physical model. Five trials were performed for each, and the average values are shown.

The relationships between *d*_*b*_ and *d*_*h*_ and the relationships between *d*_*b*_ and *d*_*p*_ are shown in Figure 12(c). For *d*_*b*_ = 20,30,40 mm, *d*_*h*_ and *d*_*p*_ had an error of less than 5 mm, which is accurate enough for the inserting motion. For *d*_*b*_ = 10 mm, the deformation was too small to have an error, and for *d*_*b*_ = 50 mm, the deformation was so large as to exceed the limits of the model, causing an error. These results show that the estimated values are sufficiently reliable for appropriate transformations. Note that in Figure 12(d), the estimated values of *d*_*p*_ are all 80 mm because the model points are placed every 10 mm.

### 6.3. Valley Folds Twice in a Row

We tried two kinds of experiments [7]. In one experiment, the hand executed diagonal valley folds twice on a flat sheet of paper, and in the other, the hand executed only the second valley fold on a sheet of paper folded diagonally by a human beforehand. The distance between the two corners C and D was used to evaluate the accuracy.

In this experiment, *λ* = 0.05, *h* = 0.084, and *k* = 0.3 were used. These hyperparameters were determined empirically based on experiments. Since these parameters depend on the paper's characteristics, they need to be adjusted according to the object. We verified that these parameters were at least effective for the same types of sheets of paper.

Table 5 shows the success rate and the distance error. In the case of only the second valley fold, all ten folds were successful, and in the case of the first and second valley folds, 9 out of 10 folds were successful. In the former, the minimum and average errors were 2.5 mm and 5.25 mm, respectively, and in the latter, the minimum and average errors were 4.5 mm and 9.72 mm, respectively.

**Figure 13:**
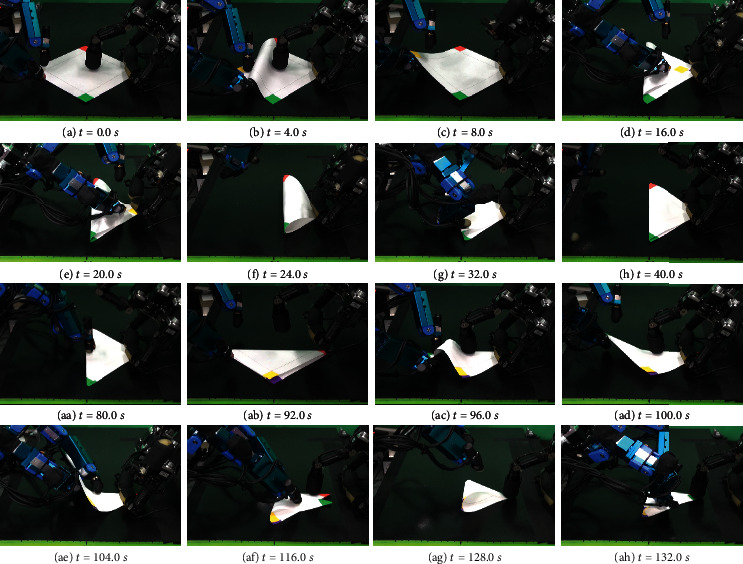
Paper folding motion of 1st and 2nd valley folds.

Figure 13 shows continuous photographs of one folding behavior. The first folding is completed from (a) to (h), and the second folding is completed from (aa) to (ah).

#### 6.3.1. Motion Correction in Primitive 3 (Turning-Up)

**Figure 14:**
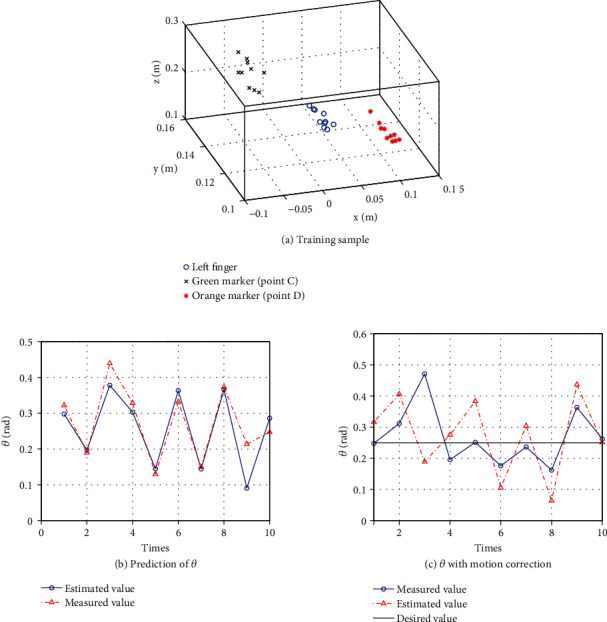
Motion correction in Primitive 3 ((b) and (c) are cited from [7]).

The algorithm for the motion correction proposed in Section 5.3 was verified. The number of training samples was 20. The data of the training sample is shown in Figure 14(a).

The predicted value of *θ* before the turning-up and the actual value of *θ* after the turning-up were measured 10 times. Figure 14(b) shows that the predicted value and the measured value were almost identical. On the 9th attempt, the prediction was far off because the paper got caught in the wiring of the hand, which disrupted the folding motion.

We also performed 10 trials with the target value of *θ*_ref_ = 0.25[rad] for the turning-up when the correction law in Eq. (12) was used. Figure 14(c) shows the predicted value of *θ* and the actual measured value after the turning-up with the correction. Except for the third trial, the variation of the measured values after the turning-up was smaller than the predictions before the turning-up due to the correction law. The prediction failed because the finger slipped significantly on the third trial.

### 6.4. Squash Fold

We performed 10 trials of the squash fold only and measured the distance between the vertices C and A (=B) after the fold. The results are shown in Table 6. Folding succeeded 8 times out of the 10 trials.

**Figure 15:**
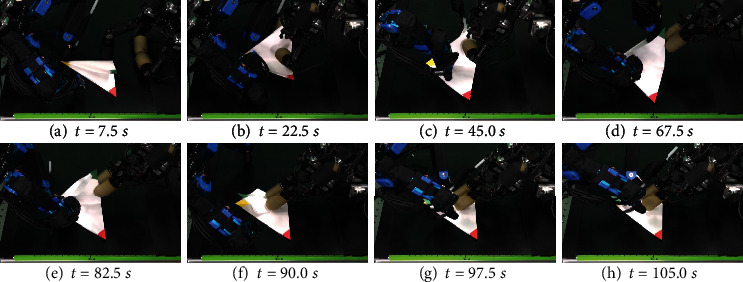
Squash folding motion.

The results are shown in the series of pictures in Figure 15. From Table 6, the error's minimum value was 2.28 mm, while the average value was 15.51 mm, indicating that stable folding could not be achieved. The reason is that slippage occurred when the paper was crushed. At this phase, the physical simulator was not sufficient either, as the occlusion region was so large that the corners could not be visually measured.

## 7. Conclusion

In this paper, we proposed a new general-purpose robot system with sufficient capabilities for performing Origami. The system has a two-fingered hand with redundant fingers for dexterous manipulation, a three-fingered hand, a dual-arm with linear sliders, a depth sensor for measuring the paper's state, and a physical simulator for simulating the sheet of paper. The complex folding processes are decomposed into simple motion primitives, which increased the success rate. To estimate the paper deformation in real-time, we use a physical simulator in which some quality points are connected by springs, and the depth sensor updates the model. As a result, we achieved consecutive valley folds and a squash fold.

One of the most critical problems with the current system is the need to estimate the shape of the sheet of paper. In the present configuration, the system observes only some feature points, and the physical simulation model helps to achieve better estimation. However, this is not sufficient for recognizing the whole shape and precise conditions of the sheet, and we are currently investigating accurate registration of the sheet of paper by integrating 3D information and a physical model [20, 21].

## Figures and Tables

**Table 1:** Specifications of left two-redundant-fingered hand.

Joint number	1	2	3	4	5	6	7
Reduction rate	50	300	100	150	100	90	100
Max. torque [Nm]	0.29	0.35	0.44	0.17	0.11	0.1	0.11
Actuator type	RSF-5A	AU	RSF-5A	AU	RSF-3B	AU	RSF-3B

**Table 2:** Specifications of right three-fingered hand.

Joint number	1 (palm)	2 (root)	3 (tip)	4 (tip rotation)
Reduction rate	50	50	100	50
Max. torque [Nm]	0.7	0.7	0.29	0.29
Actuator type	RSF-5A	RSF-5A	RSF-3B	RSF-3B

**Table 3:** Specifications of dual-arm.

Joint number	1 (*X*)	2 (*Y*)	3 (*Z*)	Joint number	4	5	6
Screw lead [mm]	10	10	5	Reduction rate	50	50	50
Max. force [N]	155	155	302	Max. torque [Nm]	18	1.5	0.36
Actuator type	HF-KP13B			Actuator	FHA-14C	FHA-8C	CSF-8

**Table 4:** Success rate and error in first valley-fold.

Times	Success	Max [mm]	Min [mm]	Average [mm]
10	10	5.80	0.54	2.70

**Table 5:** Success rate and error in second valley fold (cited from [7]).

	Times	Success	Max [mm]	Min [mm]	Ave [mm]
2nd valley-fold	10	10	8.50	2.50	5.25
1st and 2nd valley-folds	10	9	19.0	4.50	9.72

**Table 6:** Success rate and distance error in squash folding.

Times	Success	Max [mm]	Min [mm]	Ave [mm]
10	8	28.97	2.28	15.51

## Data Availability

The data used to support the findings of this study are available from the corresponding author upon request.

## Supplementary Materials

Supplementary Materials This video shows the results of consecutive valley folds and a squash fold.
